# Genetic Polymorphisms in Toll-Like Receptors among Pediatric Patients with Renal Parenchymal Infections of Different Clinical Severities

**DOI:** 10.1371/journal.pone.0058687

**Published:** 2013-03-04

**Authors:** Chi-Hui Cheng, Yun-Shien Lee, Chee-Jen Chang, Tzou-Yien Lin

**Affiliations:** 1 Division of Pediatric Nephrology, Department of Pediatrics, Chang Gung Children’s Hospital, Chang Gung Memorial Hospital, Taoyuan, Taiwan; 2 College of Medicine, Chang Gung University, Taoyuan, Taiwan; 3 Genomic Medicine Research Core Laboratory (GMRCL), Chang Gung Memorial Hospital, Taoyuan, Taiwan; 4 Department of Biotechnology, Ming-Chuan University, Taoyuan, Taiwan; 5 Statistical Center for Clinical Research, Chang Gung Memorial Hospital, Taoyuan, Taiwan; 6 Division of Pediatric Infectious Diseases, Department of Pediatrics, Chang Gung Children’s Hospital, Chang Gung Memorial Hospital, Taoyuan, Taiwan; Wayne State University School of Medicine, United States of America

## Abstract

**Background:**

Although several studies have suggested single gene defects or variations in the genes associated with host immune response could confer differences in susceptibility to urinary pathogen invasion, no studies have examined the genetic polymorphisms in various toll-like receptors (TLRs) that activate innate immune responses in pediatric renal parenchymal infections of different clinical severities, namely acute pyelonephritis and the clinically more severe disease, acute lobar nephronia.

**Methodology:**

Patients who fulfilled the diagnostic criteria for acute pyelonephritis (APN) and acute lobar nephronia (ALN) without underlying diseases or structural anomalies, except for vesicoureteral reflux (VUR), were enrolled. Genotyping of the single nucleotide polymorphisms (SNPs) in the genes encoding TLR-1, TLR-2, TLR-4, TLR-5, and TLR-6 was performed by matrix-assisted laser desorption/ionization time-of-flight-based mini-sequencing analysis.

**Principal Findings:**

A total of 16 SNPs were selected for genotyping. Analysis of 96 normal and 48 patients’ samples revealed that only four SNPs had heterozygosity rates >0.01. These SNPs were selected for further investigation. Hardy-Weinberg equilibrium was satisfied for the observed genotype frequencies. Statistically significant differences in the genotype frequency of *TLR-2* (rs3804100, T1350C) between controls and ALN or (APN+ALN) combined group were identified using the recessive model with the correction for multiple-SNP testing. Further genotype pattern frequency analysis in *TLR-2* SNPs (rs3804099 and rs3804100) showed significantly reduced occurrence of the rare allele homozygote (CC+CC) in the no-VUR subgroup of APN and ALN cases.

**Conclusions:**

As the inflammatory responses in ALN patients are more severe than those in APN patients (higher CRP levels, longer duration of fever after antibiotic treatment), these findings suggest that the genetic variant in *TLR-2* (rs3804100, T1350C) may protect the host from severe urinary tract infections as ALN.

## Introduction

Urinary tract infections (UTIs) are among the most prevalent infectious bacterial diseases in infants and children. The morbidity risk was estimated to be approximately 3% in prepubertal girls, 1% in prepubertal boys, and 8% in girls [1]. The clinical severity of UTIs ranges from uncomplicated lower urinary tract infections to frank abscess formation. Among the UTIs, acute lobar nephronia (ALN), also known as acute focal bacterial nephritis, presents as a localized nonliquefactive inflammatory renal bacterial infection and has previously been identified as a complicated form of acute renal infection, representing progression of the inflammatory process of acute pyelonephritis (APN) [2]. ALN may also represent a relatively early stage in renal abscess development [3]. It is generally accepted that renal parenchymal infections, including APN, ALN, and intrarenal abscess formation, are the more serious forms of UTI and have a longer duration of antibiotic treatment. Moreover, in some cases, surgical procedures are recommended for proper management [2], [4], [5].

Complex host-pathogen interactions determine patient susceptibility to UTIs and clinical severity. A number of studies have demonstrated that certain virulence factors associated with the uropathogenic bacterium Escherichia coli, a common clinical isolate, are more prevalent in specific UTIs [4], [6]. Nevertheless, intra-individual variation in clinical presentation has been noted among UTI patients. This indicates that host factors such as mechanistic dysfunction [e.g., vesicoureteral reflux (VUR)] and genetic variation in the susceptibility to bacterial invasion and infection should not be overlooked [7]–[9].

The innate immune system has been recognized as the first line of defense against invading pathogens and plays a primary role in acute host defense [10]. Variations in genes that modulate innate immune responses may result in distinct clinical presentations in UTIs. Among these genes are those encoding Toll-like receptors (TLRs), which recognize pathogen-associated molecular patterns (PAMPs), and those encoding chemokines and chemokine receptors, which facilitate the migration of neutrophils to the infected urinary tract. Single gene defects or variations in these genes could confer differences in susceptibility to urinary pathogen invasion [7]–[9], [11]–[13].

Escherichia coli, the most common uropathogen in renal parenchymal infections [4], [5], is recognized by various TLRs, including TLR-1, TLR-2, TLR-4, TLR-5, TLR-6 (in humans and mice), and TLR-11 (in mice) [7], [11], [14], [15]. Previous studies have shown that single nucleotide polymorphisms (SNPs) in the TLR-2 and TLR-4 genes can affect host susceptibility to UTIs [7], [11], [13], [16]–[19]. In contrast, we did not observe this association for TLR-4 in APN and ALN [12].

To extend our previous analysis of genetic polymorphisms in pediatric patients with renal parenchymal infections [12], this study explored the correlations between polymorphisms in UTI-related TLR genes (TLR-1, TLR-2, TLR-4, TLR-5, and TLR-6) and clinical severity among pediatric patients with UTIs of different severities (APN and the clinically more severe disease, ALN). In addition, as VUR is a well-known risk factor for severe parenchymal infectious disease [8], [20], a subgroup of APN and ALN patients without VUR was also examined to exclude the possible effects of VUR.

## Materials and Methods

### Ethics Statement

This investigation was approved by the Institutional Review Board of Chang Gung Memorial Hospital, and following a full explanation of the study, written informed consent was obtained from the parents of all patients.

### Study Setting and Patient Selection Criteria

This study is a part of our continuing analyses of the pathogenic host and bacterial urovirulence factors related to APN and ALN [4], [5], [12]. The participating patients were admitted to Chang Gung Children’s Hospital, a tertiary medical center located in a suburb of Taipei in northern Taiwan, between January 2004 and December 2008. Patients who fulfilled the diagnostic criteria for APN and ALN caused by *E. coli* while lacking any of the exclusion criteria were enrolled in the study. The controls were pediatric patients who presented to the outpatient clinic for reasons other than a UTI or severe infection and were interviewed to ensure that they did not have a history of UTI or severe infections as well as not having positive urine culture.

The diagnostic scheme for patients suspected of having APN or ALN was as described previously [2], [4], [5], [12]. In brief, all patients with a suspected UTI because of the presence of pyuria (>5 white blood cells/high-power field) and fever with symptoms and signs related to UTIs (*e.g.*, pain, dysuria, and frequency of urination) or without focus underwent renal ultrasonography on the first or second day after admission. Computed tomography (CT) was performed immediately when the initial ultrasonographic findings met either of two criteria: evidence of unilateral or bilateral nephromegaly or a focal renal mass. For children who presented with borderline nephromegaly on ultrasonography, CT was performed when the child remained febrile for 72 h after the commencement of antibiotic therapy. A diagnosis of ALN was made on the basis of positive CT findings. Technetium 99m-dimercaptosuccinic acid scintigraphy (^99m^Tc-DMSA) was performed within 3–7 days of admission in patients suspected of having a febrile UTI who did not satisfy the sonographic criteria for ALN. APN was defined as focal or diffuse areas of decreased ^99m^Tc-DMSA uptake without evidence of cortical loss.

Patients with evidence of an underlying disease, including diabetes and immunodeficiency, or structural anomalies such as neurogenic bladder, posterior urethral valve, urinary diversion, bladder diverticulum, ureterocele, or urinary tract obstruction other than VUR were excluded.

### Genotyping by Matrix-assisted Laser Desorption/ionization Time-of-flight (MALDI-TOF)-based Mini-sequencing Analysis

**Figure 1:**
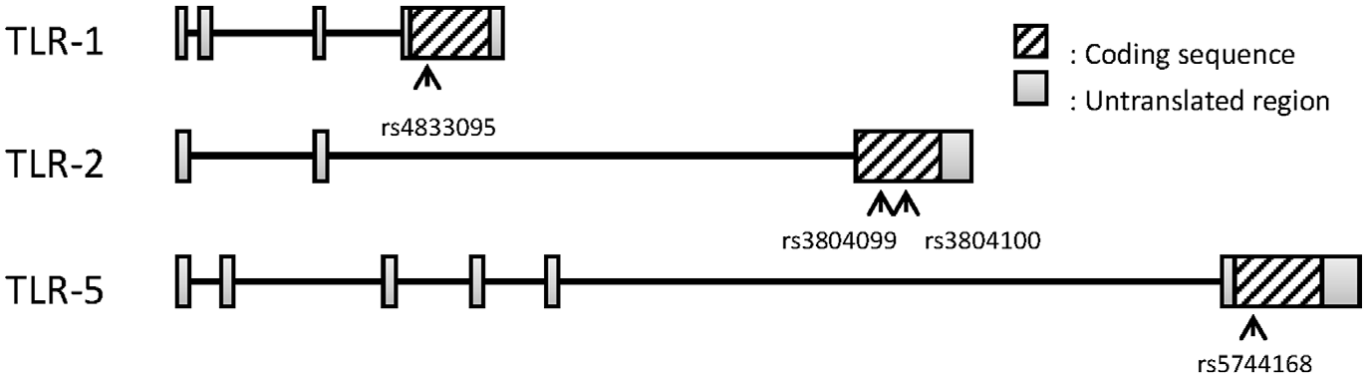
Position of the four SNPs that had heterozygosity rates >0.01 in each TLR sequence.

SNPs in the genes encoding TLR-1, TLR-2, TLR-4, TLR-5, and TLR-6, as well as in their respective promoter regions, were identified in the NCBI dbSNP database [21]. A total of 16 SNPs (rs4833095, rs5743611, and rs5743618 for *TLR-1*; rs3804099, rs3804100, rs5743704, and rs5743708 for *TLR-2*; zA11547G, rs2149356, and rs5030710 for *TLR-4*; rs2072493, rs5744168, and rs5744174 for *TLR-5* and rs3821985, rs5743810 and rs5743815 for *TLR-6*) were selected for genotyping based on previous studies on Toll-like receptors polymorphisms on APN adult patients [7], [11]. Analysis of 96 normal and 48 patients’ samples revealed that only four SNPs had heterozygosity rates >0.01. These SNPs (Table 1, Figure 1) were used to analyze the control and patient genotypes by a MALDI-TOF-based mini-sequencing genotyping method.

**Table 1:** Primers used for the amplification and mini-sequencing analysis of the SNPs.

Position	Primer sequence (5′→3′)	PCR product size	Mini-sequencing primer sequence (5′→3′)	Molecular weight of mini-sequencing product
*TLR-1* (rs4833095), C>T	Sense: tccagctgaccctgtagctt	157 bp	caatgttgtttaaggtaaga	Primer: 6,195.06
	Anti-sense: ttctggcgaaacttcaaaca			T allele: 6,483.25
				C allele: 6,772.43
*TLR-2* (rs3804099), C>T	Sense: tgctggacttaccttccttga	182 bp	agtttgaagtcaattcagaa	Primer: 6,164.05
	Anti-sense: ctcgcagttccaaacattcc			T allele: 6,781.45
				C allele: 6,437.23
*TLR-2* (rs3804100), T>C	Sense: aaccggagagactttgctca	226 bp	agcacacgaatacacag	Primer: 5,181.42
	Anti-sense: gagttgcggcaaattcaaag			C allele: 5,454.60
				T allele: 5,798.82
*TLR-5* (rs5744168), C>T	Sense: acggacttgacaacctccaa	223 bp	tacagaccttggatctc	Primer: 5,145.36
	Anti-sense: tcgggtatgcttggaataaaa			T allele: 5,762.76
				C allele: 5,418.55

Genomic DNA was extracted from peripheral blood lymphocytes using a Nucleospin® blood kit (Macherey-Nagel, Düren, Germany) according to the manufacturer’s recommendations. The SNPs were genotyped as described previously [12] using the primers listed in Table 1. Briefly, PCR was performed in a total volume of 25 µL containing 200 ng of genomic DNA, primers (25 pM each), dNTPs (0.2 mM), 1× Fast-Start PCR buffer (50 mM Tris-HCl, 10 mM KCl, 5 mM [NH_4_]_2_SO_4_, 2mM MgCl_2_; pH 8.3), 1 M betaine, and 1 U of Fast-Start Taq Polymerase (Roche Diagnostics, Basel, Switzerland). The reaction comprised initiation at 95°C for 5 min, followed by 40 cycles of 95°C for 45 s, 50°C for 45 s, and 60°C for 45 s, with a final extension at 52°C for 10 min. Unincorporated dNTPs and primers were removed automatically by MAPIIA (GenePure PCR Purification System; Bruker, Bremen, Germany). The purified products were collected and mini-sequencing reactions were run using individual mini-sequencing primers (Table 1) in 20 µL of a solution containing 50 ng of the PCR product, 1 µL (10 pmol) of mini-sequencing primer, 0.5 µL of 1 mM ddNTP/dNTP mixture (for *TLR-1* rs4833095, dC ddT; for *TLR-2* rs3804099, dT ddC ddG; for *TLR-2* rs3804100, dT ddC ddG; and for *TLR-5* rs5744168, dT ddC ddG), 0.5 U of Thermo Sequenase DNA Polymerase (Amersham Biosciences, Piscataway, NJ), and 2 µL of the reaction buffer provided by the manufacturer. The reactions were carried out in a multiblock thermal cycler (Thermo Hybaid, Waltham, MA) with initial denaturation at 96°C for 1 min, followed by 50 cycles of 96°C for 15 s, 50°C for 15 s, 60°C for 100 s, and 96°C for 30 s. The reaction products were purified automatically by MAPIIA (Single-Strand DNA Binding Beads; Bruker) and analyzed by MALDI-TOF mass spectrometry (MS).

The samples were then mixed with 0.5 µL of matrix solution (50 mg/mL 3-hydropicolinic acid in a 4∶5∶1 mixture of water, acetonitrile, and 50 mg/mL diammonium citrate) and spotted onto 384-well Teflon sample plates (PerSeptive Biosystems, Framingham, MA). MALDI-TOF mass spectra were acquired with a Bruker Autoflex MALDI-TOF mass spectrometer (Bruker) and AutoXecute software (Bruker) to validate the genotype data. To confirm the MALDI-TOF analysis results, 10% of the PCR products were randomly selected for auto-sequencing analysis using an ABI 3730 autosequencer (Applied Biosystems, Foster City, CA).

### Statistical Analysis

Hardy-Weinberg equilibrium was tested for goodness-of-fit using a χ^2^ test with one degree of freedom, to compare the observed and expected genotype frequencies among the study subjects. The association of case-control status (outcome) and SNP genotype was analyzed using log-additive (or allelic-trend test), recessive, and dominant models [7], [11]. In the log-additive model, common homozygous genotypes (00) were assigned a value of 0; heterozygotes (01), a value of 1; and minor homozygous genotypes (11), a value of 2. For the dominant model, the genotypes 01 and 11 were combined and compared with genotype 00. In a similar manner, the genotypes 00 and 01 were combined and compared with genotype 11 for recessive model analysis. Odds ratios and significance levels were assessed using a logistic regression model. Statistical comparisons of categorical variables or binominal results (*e.g.*, allele frequency) among the control, APN, and ALN groups were performed by χ^2^ analysis or two-sided Fisher’s exact test, as appropriate. Genotype pattern frequency analysis for the SNPs with heterozygosity rates >0.01 in *TLR-2* (i.e. rs3804099, rs3804100) was also performed using a similar method as described by Ragnarsdóttir et al [17]. All statistical analyses were performed using SPSS software (Version 16.0, IBM SPSS Statistics) or otherwise, the website tools as stated.

At first, 96 cases (48 APN and 48 ALN) and 96 control samples were analyzed for *TLR-2* (rs3804100) SNP. The prior data (192 samples) indicated that the probability of exposure among controls is 0.10417. With the recessive model, if the true odds ratio for disease in exposed subjects relative to unexposed subjects is 0.182979 as we noted among these 192 samples, we will need to study 154 patients and 154 control patients to be able to reject the null hypothesis that this odds ratio equals 1 with probability (power) of 0.8. The Type I error probability associated with this test of this null hypothesis is 0.05. The samples size estimate was performed with PS Power and Sample Size Calculations Version 3.0 (http://biostat.mc.vanderbilt.edu/PowerSampleSize) [22].

The demographic and clinical characteristics of the patients enrolled were described in a previous publication [12]. All cases are Taiwanese. The gender ratio and age were not significantly different (*p*>0.05) among the control patients (*n* = 222), APN patients (*n = *113), and ALN patients (*n = *172). In addition, patients with ALN (the clinically more severe UTI) presented with a significantly higher C-reactive protein (CRP) level than the APN patients. Moreover, the durations of fever prior to admission and after antibiotic treatment were longer in the ALN patients than in the APN patients. However, no statistically significant difference in white blood cell count was noted.

## Results

**Figure 2:**
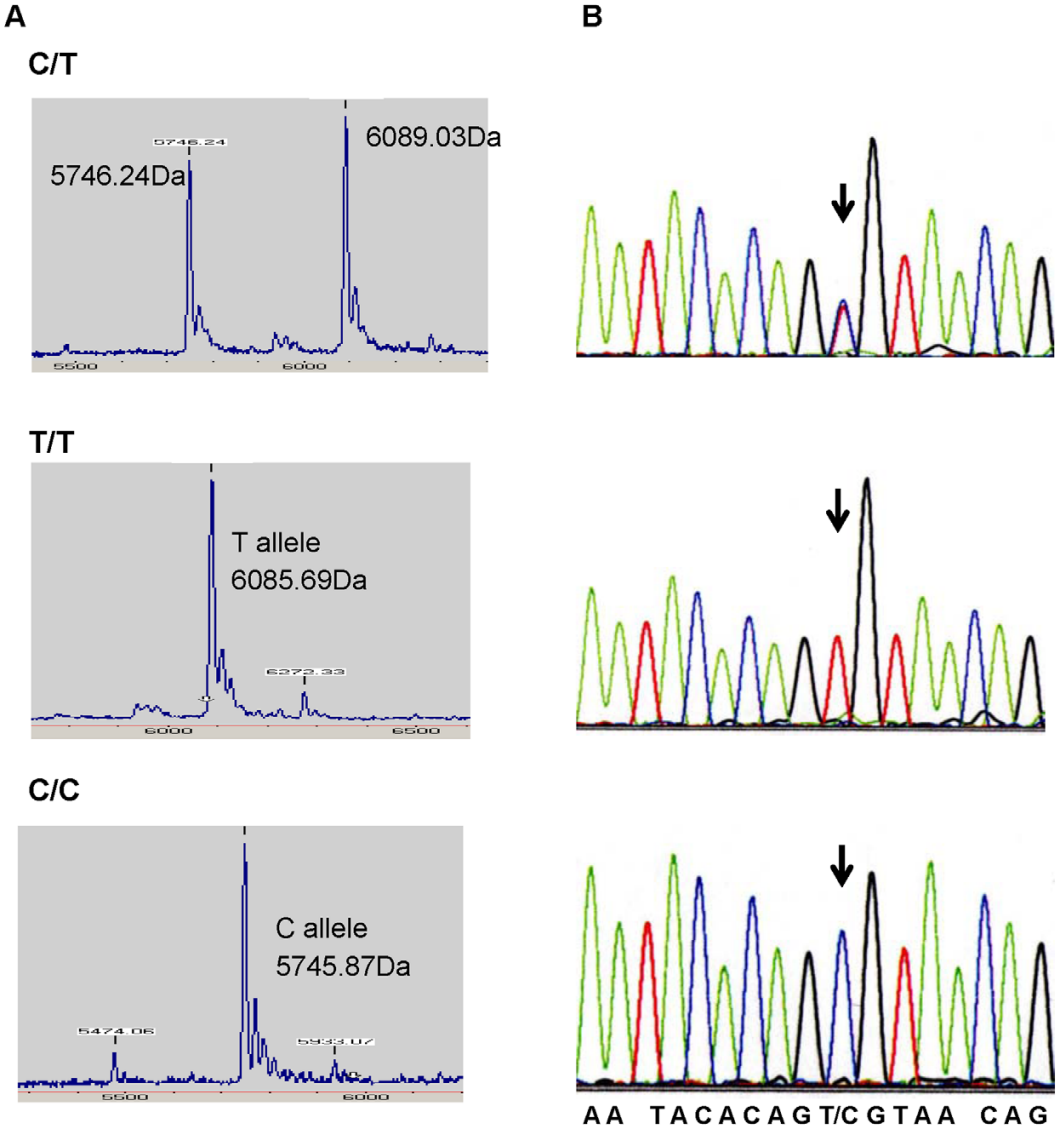
MALDI-TOF mass spectra from the genotyping of *TLR-2* (rs3804100) PCR product and its sequencing results. (A) The SNPs were genotyped by MALDI-TOF MS based on the molecular weights of the mini-sequencing products listed in Table 1. (B) Sequencing results for each of the PCR products from the C/T, T/T, and C/C genotypes of rs3804100. The SNPs are indicated by arrowheads.

Hardy-Weinberg equilibrium was satisfied in the observed genotype frequencies for all groups (Table 2). Auto-sequencing of randomly selected PCR products, for example, *TLR-2* (rs3804100) for three enrolled individuals, gave results that were identical to those derived from the MALDI-TOF mass spectra (Figure 2). This indicates the MALDI-TOF method was able to precisely determine the DNA sequences of the SNPs.

**Table 2:** Genotypic analysis of the SNPs.

SNP	Group	Genotype, *n* (%)			Log-additive model		Dominant model		Recessive model	
		00	01	11			(01, 11 *vs.* 00)		(11 *vs.* 00, 01)	
		CC	CT	TT	OR (95% CI)	*P* a	OR (95% CI)	*P* a	OR (95% CI)	*P* a
*TLR-1*	Control	75 (34.1)	113 (51.4)	32 (14.5)			1.00		1.00	
(rs4833095)	APN	44 (40.7)	51 (47.2)	13 (12.0)	0.81 (0.57, 1.15)	0.243	0.75 (0.47, 1.21)	0.241	0.80 (0.40, 1.69)	0.531
	ALN	59 (35.1)	81 (48.2)	28 (16.7)	1.02 (0.76, 1.37)	0.876	0.96 (0.63, 1.46)	0.833	1.17 (0.68, 2.04)	0.568
	Combined^b^	103 (37.3)	132 (47.8)	41 (14.9)	0.94 (0.72, 1.22)	0.635	0.87 (0.60, 1.26)	0.456	1.02 (0.62, 1.69)	0.923
		**TT**	**TC**	**CC**	**OR (95% CI)**	***P***	**OR (95% CI)**	***P***	**OR (95% CI)**	***P***
*TLR-2*	Control	102 (46.8)	97 (44.5)	19 (8.7)			1.00		1.00	
(rs3804099)	APN	47 (43.5)	57 (52.8)	4 (3.7)	0.95 (0.66, 1.39)	0.810	1.14 (0.72, 1.82)	0.577	0.40 (0.13, 1.22)	0.080
	ALN	72 (43.4)	81 (48.8)	13 (7.8)	1.07 (0.77, 1.47)	0.698	1.15 (0.76, 1.72)	0.505	0.89 (0.43, 1.86)	0.756
	Combined	119 (43.4)	138 (50.4)	17 (6.2)	1.02 (0.77, 1.36)	0.880	1.15 (0.80, 1.64)	0.457	0.69 (0.35, 1.37)	0.290
		**TT**	**TC**	**CC**	**OR (95% CI)**	***P***	**OR (95% CI)**	***P***	**OR (95% CI)**	***P***
*TLR-2*	Control	112 (50.9)	92 (41.8)	16 (7.3)			1.00		1.00	
(rs3804100)	APN	53 (48.6)	54 (49.5)	2 (1.8)	0.91 (0.62, 1.35)	0.652	1.10 (0.69, 1.73)	0.696	0.24 (0.05, 1.06)	**0.026**
	ALN	83 (50.0)	80 (48.2)	3 (1.8)	0.88 (0.62, 1.24)	0.451	1.04 (0.69, 1.55)	0.860	0.23 (0.07, 0.82)	**0.009** c
	Combined	136 (49.5)	134 (48.7)	5 (1.8)	0.89 (0.65, 1.21)	0.444	1.06 (0.74, 1.51)	0.748	0.24 (0.09, 0.66)	**0.003** c
		**CC**	**CT**	**TT**	**OR (95% CI)**	***P***	**OR (95% CI)**	***P***	**OR (95% CI)**	***P***
*TLR-5*	Control	210 (95.5)	10 (4.5)	0 (0.0)						
(rs5744168)	APN	100 (92.6)	8 (7.4)	0 (0.0)	1.68 (0.64, 4.39)	0.296				
	ALN	153 (93.9)	10 (6.1)	0 (0.0)	1.37 (0.56, 3.38)	0.492				
	Combined	253 (93.4)	18 (6.6)	0 (0.0)	1.49 (0.68, 3.31)	0.315				

a *P* values <0.05 are shown in bold.

b APN+ALN.

c Statistical significance with correction for multiple-SNP testing (*P*<0.0125).

Statistical analyses revealed that only the *TLR-2* (rs3804100) SNP showed a significant difference in genotype frequency between the control group and the APN and ALN groups (and a combined APN+ALN group) using the recessive model [OR (95% CI): APN *vs*. control, 0.24 (0.05, 1.06); ALN *vs*. control, 0.23 (0.07, 0.82); APN+ALN *vs*. control, 0.24 (0.09, 0.66)] (Table 2). After correction for multiple-SNP testing (4 SNPs examined here), only ALN and (APN+ALN) groups showed significant difference *vs*. control in recessive model (*P*<0.0125). There were no statistically significant differences in the allele frequencies of the SNPs we examined (Table 3).

**Table 3:** Allele frequency analysis of the SNPs using the logistic regression model.

SNP	T allele frequency (%)				APN *vs.* control		ALN *vs.* control		Combined^a^ *vs.* control	
	Control	APN	ALN	Combined^a^	OR (95% CI)	*P*	OR (95% CI)	*P*	OR (95% CI)	*P*
*TLR-1* (rs4833095)	40.23	35.65	40.77	38.77	0.82 (0.59, 1.15)	0.257	1.02 (0.77, 1.37)	0.878	0.94 (0.73, 1.22)	0.640
*TLR-2* (rs3804099)	69.04	69.91	67.77	68.61	1.04 (0.73, 1.49)	0.820	0.94 (0.69, 1.28)	0.709	0.98 (0.75, 1.29)	0.887
*TLR-2* (rs3804100)	71.82	73.39	74.10	73.82	1.08 (0.75, 1.56)	0.670	1.12 (0.81, 1.55)	0.481	1.11 (0.83, 1.47)	0.482
*TLR-5* (rs5744168)	2.27	3.70	3.07	3.32	1.65 (0.64, 4.25)	0.303	1.36 (0.56, 3.31)	0.500	1.48 (0.67, 3.23)	0.322

a APN+ALN.

Because VUR has been suggested to be a significant host risk factor for upper UTIs [8], [20], further genetic analysis was conducted in subgroups of APN and ALN patients with no VUR (APN, 50 patients; ALN, 108 patients). As a voiding cystourethrography was not medically indicated in the control patients, the number of patients with VUR in the control group was calculated based on a reported prevalence rate [23]. Given a 0.3% prevalence rate of VUR at the mean age of the control group (2.91±3.01 years), the number of individuals with VUR among the 222 control cases was assumed to be zero. The age and gender ratios remained not significantly different (*p*>0.05) between the control group and the APN and ALN subgroups with no VUR.

In comparison with the *TLR-2* (rs3804100) TT and TC genotype frequencies, the CC genotype frequency was significantly lower in the no-VUR ALN and APN+ALN patient subgroups [recessive model, OR (95% CI): ALN *vs*. control, 0.24 (0.05, 1.07); APN+ALN *vs*. control, 0.33 (0.11, 1.01)] (Table 4). With correction for multiple-SNP testing, no significant difference was noted between the control and the no-VUR disease subgroups. The allele frequency analyses showed no significant difference between the control group and the no-VUR APN, ALN, or APN+ALN patient subgroup (Table 5).

**Table 4:** Genotypic analysis of the SNPs in the no-VUR patient subgroup.

SNP	Group	Genotype, *n* (%)			Log-additive model		Dominant model		Recessive model	
		00	01	11			(01, 11 *vs.* 00)		(11 *vs.* 00, 01)	
		CC	CT	TT	OR (95% CI)	*P* a	OR (95% CI)	*P* a	OR (95% CI)	*P* a
*TLR-1*	Control	75 (34.1)	113 (51.4)	32 (14.5)			1.00		1.00	
(rs4833095)	APN	21 (42.0)	24 (48.0)	5 (10.0)	0.75 (0.47, 1.20)	0.230	0.71 (0.38, 1.34)	0.296	0.65 (0.24, 1.77)	0.383
	ALN	33 (30.6)	55 (50.9)	20 (18.5)	1.18 (0.84, 1.65)	0.346	1.18 (0.72, 1.93)	0.521	1.34 (0.72, 2.47)	0.360
	Combined^b^	54 (34.2)	79 (50.0)	25 (15.8)	1.03 (0.76, 1.39)	0.866	1.00 (0.65, 1.53)	0.986	1.10 (0.63, 1.95)	0.733
		**TT**	**TC**	**CC**	**OR (95% CI)**	***P***	**OR (95% CI)**	***P***	**OR (95% CI)**	***P***
*TLR-2*	Control	102 (46.8)	97 (44.5)	19 (8.7)			1.00		1.00	
(rs3804099)	APN	21 (42.0)	27 (54.0)	2 (4.0)	1.00 (0.61, 1.64)	0.994	1.21 (0.65, 2.26)	0.539	0.44 (0.10, 1.94)	0.228
	ALN	46 (42.6)	56 (51.9)	6 (5.6)	1.03 (0.71, 1.49)	0.888	1.19 (0.74, 1.89)	0.473	0.62 (0.24, 1.59)	0.300
	Combined	67 (42.4)	83 (52.5)	8 (5.1)	1.02 (0.73, 1.42)	0.909	1.19 (0.79, 1.80)	0.399	0.56 (0.24, 1.31)	0.168
		**TT**	**TC**	**CC**	**OR (95% CI)**	***P***	**OR (95% CI)**	***P***	**OR (95% CI)**	***P***
*TLR-2*	Control	112 (50.9)	92 (41.8)	16 (7.3)			1.00		1.00	
(rs3804100)	APN	25 (50.0)	23 (46.0)	2 (4.0)	0.94 (0.57, 1.55)	0.806	1.04 (0.56, 1.92)	0.908	0.53 (0.12, 2.39)	0.375
	ALN	54 (50.0)	52 (48.1)	2 (1.9)	0.88 (0.60, 1.30)	0.519	1.04 (0.65, 1.64)	0.877	0.24 (0.05, 1.07)	**0.027** c
	Combined	79 (50.0)	75 (47.5)	4 (2.5)	0.90 (0.64, 1.27)	0.536	1.04 (0.69, 1.56)	0.862	0.33 (0.11, 1.01)	**0.034** c
		**CC**	**CT**	**TT**	**OR (95% CI)**	***P***	**OR (95% CI)**	***P***	**OR (95% CI)**	***P***
*TLR-5*	Control	210 (95.5)	10 (4.5)	0 (0.0)						
(rs5744168)	APN	45 (93.8)	3 (6.2)	0 (0.0)	1.40 (0.37, 5.29)	0.630				
	ALN	99 (94.3)	6 (5.7)	0 (0.0)	1.27 (0.45, 3.60)	0.653				
	Combined	144 (94.1)	9 (5.9)	0 (0.0)	1.31 (0.52, 3.31)	0.566				

a *P* values <0.05 are shown in bold.

b APN+ALN.

c Statistical non-significance with correction for multiple-SNP testing (*P*>0.0125).

**Table 5:** Allele frequency analysis of the SNPs in the no-VUR patient subgroup using the logistic regression model.

SNP	T allele frequency (%)				APN *vs.* control		ALN *vs.* control		Combined^a^ *vs.* control	
	Control	APN	ALN	Combined^a^	OR (95% CI)	*P*	OR (95% CI)	*P*	OR (95% CI)	*P*
*TLR-1* (rs4833095)	40.23	34.00	43.98	40.82	0.77 (0.49, 1.21)	0.246	1.17 (0.84, 1.62)	0.360	1.03 (0.76, 1.38)	0.869
*TLR-2* (rs3804099)	69.04	69.00	68.52	68.67	1.00 (0.62, 1.60)	0.994	0.98 (0.69, 1.39)	0.893	0.98 (0.72, 1.34)	0.915
*TLR-2* (rs3804100)	71.82	73.00	74.07	73.73	1.06 (0.65, 1.73)	0.812	1.12 (0.78, 1.62)	0.542	1.10 (0.80, 1.53)	0.560
*TLR-5* (rs5744168)	2.27	3.13	2.86	2.94	1.39 (0.37, 5.14)	0.634	1.26 (0.45, 3.53)	0.657	1.30 (0.52, 3.25)	0.571

a APN+ALN.

**Figure 3:**
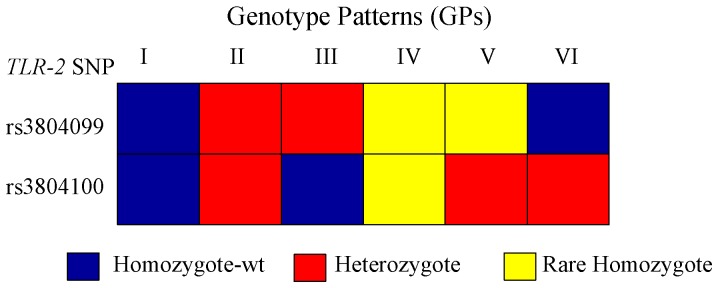
*TLR-2* genotype patterns of the SNPs with heterozygosity rates >0.01. Each column represents a genotype pattern and each row a SNP in *TLR-2*. Common allele homozygote (blue), heterozygote (red) and rare allele homozygote (yellow) are shown.

Genotype patterns in *TLR-2* were assigned by combining multiple SNPs that had heterozygosity rates >0.01 (i.e. rs3804099, rs3804100) in each individual (Figure 3) [17]. The frequency of genotype pattern IV (CC+CC) was significantly reduced in the APN+ALN combined group (Table 6). After the elimination of cases with VUR, genotype pattern IV can not even be noted in the no-VUR subgroup of APN and ALN (Table 7).

**Table 6:** Genotype pattern frequency analysis of the SNPs in *TLR-2* (rs3804099 and rs3804100) using the two-tailed Fisher’s Exact Test#.

Genotype pattern	Genotype pattern frequency (*n*)			APN *vs.* control	ALN *vs.* control	Combined^b^ *vs.* Control
	Control (218)	APN (107)	ALN (166)	*P* a	*P* a	*P* a
I (TT+TT)	99	43	70	0.406	0.535	0.409
II (TC+TC)	83	49	70	0.189	0.462	0.231
III (TC+TT)	12	8	11	0.624	0.669	0.578
IV (CC+CC)	12	2	3	0.156	0.108	**0.044**
V (CC+TC)	7	2	8	0.723	0.438	0.810
VI (TT+TC)	1	3	2	0.106	0.581	0.234
Others	4	0	2	0.307	0.702	0.414

a *P* values <0.05 are shown in bold.

b APN+ALN.

# 2×2 contingency table, http://www.vassarstats.net/tab2×2.html.

**Table 7:** Genotype pattern frequency analysis of the SNPs in *TLR-2* (rs3804099 and rs3804100) in the no-VUR patient subgroup using the two-tailed Fisher’s Exact Test#.

Genotype pattern	Genotype pattern frequency (*n*)			APN *vs.* control	ALN *vs.* control	Combined^b^ *vs.* Control
	Control (218)	APN (50)	ALN (108)	*P* a	*P* a	*P* a
I (TT+TT)	99	19	42	0.350	0.286	0.206
II (TC+TC)	83	20	47	0.872	0.400	0.455
III (TC+TT)	12	6	9	0.116	0.344	0.159
IV (CC+CC)	12	0	0	0.131	**0.023**	**0.002**
V (CC+TC)	7	2	3	1.000	1.000	1.000
VI (TT+TC)	1	1	2	0.339	0.256	0.314
Others	4	2	5	0.597	0.164	0.214

a *P* values <0.05 are shown in bold.

b APN+ALN.

# 2×2 contingency table, http://www.vassarstats.net/tab2×2.html.

## Discussion

Successful defense against bacterial infection requires well-integrated host innate and adaptive immune responses. The initial pathogen recognition process is mediated by the coordinated actions of various TLRs, which are located on the cell surface or within organelles such as phagosomes, and PAMPs, including bacterial flagellin, lipopolysaccharide, and bacterial lipopeptides [7], [10]–[12]. Following this microbe-sensing step, sequential activation of the immune system leads to cytokine release, recruitment of neutrophils to the site of infection, phagocytosis, and the release of free radicals [16]. The cytokines that are released play essential roles in the host innate immune response and activation of the adaptive immune system [24]. These responses determine the balance between health and disease severity [8]. Defective signal transmission in the immune response due to, for example, genetic polymorphisms in receptors and cytokines, influences an individual’s risk for infectious diseases 7,9,11,12,16,19.

TLRs are crucial for the recognition of microbes by the innate immune system and for bridging the innate and acquired immune responses [16]. In addition to acting as critical sensors of microbial attack, TLRs also serve as effectors of TLR-dependent innate defense, which enables the host to eliminate pathogens that would otherwise cause disease morbidity or mortality [17]. Many studies have suggested that SNPs in TLR genes can affect an individual’s ability to respond to TLR ligands, leading to altered susceptibility to infections or inflammation [25]. This altered susceptibility can be either a reduced inflammatory response, as occurs in asymptomatic bacteriuria, protection against pyelonephritis, and recurrent UTIs [11], [17], [19], or an exaggerated immune response that results in a severe infection, as occurs in tuberculosis and severe atopic dermatitis [26], [27].

The APN and ALN patients in this study had a lower TLR-2 (rs3804100) CC genotype frequency compared with the controls. No differences in the other TLR SNPs examined were noted among the APN, ALN, and control groups. Furthermore, after the elimination of patients with VUR, a well-known risk factor for severe UTI, from the analysis, patients with ALN, but not those with APN, had a lower TLR-2 (rs3804100) CC genotype frequency. With the correction for multiple-SNP testing, only the ALN and (APN+ALN) combined group that include VUR diagnosis showed significant reduced TLR-2 (rs3804100) CC genotype frequency compared with the controls. Further, the genotype pattern frequency analysis for the TLR-2 has shown the genotype pattern of CC+CC (rs3804099+rs3804100) was not even noted in the no-VUR subgroup of patients with APN and ALN. As the inflammatory responses in ALN patients are more severe than those in APN patients (higher CRP levels, longer fever duration after antibiotic treatment), these findings suggest that the genetic variant in TLR-2 (rs3804100, T1350C) may protect the host from severe urinary tract infections as ALN.

Among the TLRs that have been described, TLR-2 was the first human TLR to be described in host defense against gram-negative bacteria [28] but subsequent studies have demonstrated that TLR-4 is the receptor for lipopolysaccharide [16], [29]. TLR-2 polymorphisms have also been linked to various severe infections, including tuberculosis, leprosy, and septic shock [10], [30], [31]. A few studies have reported associations between UTIs and TLR-2 polymorphism at the TLR-2 (rs5743708; G2258A; Arg753Gln) site [11], [16]. The current study found that another TLR-2 genetic variant, TLR-2 (rs3804100; T1350C; Ser450Ser), can also change the host risk to severe UTIs (APN vs. ALN).

The TLR-2 (rs3804100; T1350C; Ser450Ser) SNP described here did not induce an amino acid change, and the molecular mechanism by which this synonymous polymorphism affects host susceptibility to severe UTIs is not fully understood. Many studies have provided evidence that synonymous SNPs lead to changes in protein amount, structure, and/or function via alterations in mRNA structure and stability, kinetics of translation, and alternative splicing. Moreover, synonymous SNPs may be proxies for other polymorphisms that have not been examined [32]. Further investigations of phenotypes or functional assessment of synonymous SNPs, such as using the luciferase reporter assay or gene knock-out mice [9], [17], [19], are warranted to properly determine their effects on TLR-2 expression.

As the statistical power to detect significant associations with rare genetic variants is determined based on sample size, the major limitation of this study is the small population of individuals with the CC genotype of TLR-2 (rs3804100, T1350C). A large cohort study is recommended to replicate and validate the associations of SNPs with the severe UTIs, APN and ALN.
